# Physical frailty identification using machine learning to explore the 5-item FRAIL scale, Cardiovascular Health Study index, and Study of Osteoporotic Fractures index

**DOI:** 10.3389/fpubh.2024.1303958

**Published:** 2024-05-09

**Authors:** Chen-Cheng Yang, Po-Hong Chen, Cheng-Hong Yang, Chia-Yen Dai, Kuei-Hau Luo, Tzu-Hua Chen, Hung-Yi Chuang, Chao-Hung Kuo

**Affiliations:** ^1^Graduate Institute of Medicine, College of Medicine, Kaohsiung Medical University, Kaohsiung City, Taiwan; ^2^Department of Occupational and Environmental Medicine, Kaohsiung Municipal Siaogang Hospital, Kaohsiung Medical University, Kaohsiung City, Taiwan; ^3^Department of Occupational and Environmental Medicine, Kaohsiung Medical University Hospital, Kaohsiung Medical University, Kaohsiung City, Taiwan; ^4^Department of Electronic Engineering, National Kaohsiung University of Science and Technology, Kaohsiung City, Taiwan; ^5^Department of Information Management, Tainan University of Technology, Tainan, Taiwan; ^6^Department of Family Medicine, Kaohsiung Municipal Ta-tung Hospital, Kaohsiung Medical University, Kaohsiung City, Taiwan; ^7^Research Center for Precision Environmental Medicine, Kaohsiung Medical University, Kaohsiung City, Taiwan; ^8^Department of Public Health and Environmental Medicine, Kaohsiung Medical University, Kaohsiung City, Taiwan; ^9^Department of Internal Medicine, Kaohsiung Municipal Siaogang Hospital, Kaohsiung Medical University, Kaohsiung City, Taiwan

**Keywords:** machine learning, physical frailty, model, prediction, XGBoost

## Abstract

**Background:**

Physical frailty is an important issue in aging societies. Three models of physical frailty assessment, the 5-Item fatigue, resistance, ambulation, illness and loss of weight (FRAIL); Cardiovascular Health Study (CHS); and Study of Osteoporotic Fractures (SOF) indices, have been regularly used in clinical and research studies. However, no previous studies have investigated the predictive ability of machine learning (ML) for physical frailty assessment. The aim was to use two ML algorithms, random forest (RF) and extreme gradient boosting (XGBoost), to predict these three physical frailty assessment models.

**Materials and methods:**

Questionnaires regarding demographic characteristics, lifestyle habits, living environment, and physical frailty assessment were answered by 445 participants aged 60 years and above. The RF and XGBoost algorithms were used to assess their scores for the three physical frailty indices. Furthermore, feature importance and Shapley additive explanations (SHAP) were used to determine the important physical frailty factors.

**Results:**

The XGBoost algorithm obtained higher accuracy for predicting the three physical frailty indices; the areas under the curve obtained by the XGBoost algorithm for the 5-Item FRAIL, CHS, and SOF indices were 0.84. 0.79, and 0.69, respectively. The feature importance and SHAP of the XGBoost algorithm revealed that systolic blood pressure, diastolic blood pressure, age, and body mass index play important roles in all three physical frailty models.

**Conclusion:**

The XGBoost algorithm has a more accurate predictive rate than RF across all three physical frailty assessments. Thus, ML can be a useful tool for the early detection of physical frailty.

## Introduction

Physical frailty has become an important issue in the geriatric population of super-aging societies. It is a condition wherein susceptibility to stressors increases, especially in the older adults population, (1) resulting in undesirable health consequences, such as falling, stroke, disability, hospitalization, institutionalization, and death (2–5). The prevalence of physical frailty ranges from 3.9–51.4%, (6–8) influenced by different nationalities, socioeconomic conditions, and, most importantly, the assessment tool. Currently, there is no gold-standard diagnostic tool for assessing physical frailty. Several assessments have been established, including Fried’s phenotype model (9) and the physical frailty index in Rockwood’s cumulative deficit model (10). These assessments help identify persons with physical frailty who are at high risk of adverse consequences and provide an opportunity to counteract the evolution of adverse sequelae (11).

Machine learning (ML), a subset of artificial intelligence (AI), is a method of self-learning to provide solutions (12, 13). According to scholars such as Arthur Samuel, ML provides computers with the ability to learn without explicit programming. Therefore, ML can be classified as a computer science (14). Nevertheless, ML algorithms can be classified as “supervised” or “non-supervised” (15). Supervised ML involves training the model on predictions of relationships between features and outputs from data, whereas non-supervised ML involves searching for relevant structures within a dataset (15). The advantage of supervised ML is that it can achieve a high classification rate using a large amount of labeled data (16). Random forest (RF), initially published by Breiman, is a non-parametric learning algorithm wherein classification results are determined through voting on multiple decision trees (17). It has the advantage of reducing outliers and is less susceptible to overfitting, resulting in higher classification accuracy in many applications (18). RF is widely used in mass spectrometry, soil mapping, eye-state estimation, and remote sensing imaging (19). The extreme gradient boosting (XGBoost) algorithm, proposed by Chen, (20) randomly selects subsets to iteratively fit a single predictor and obtain a minimized loss function, and introduces a stochastic gradient boosting procedure. Through regularization, Boost can reduce the risk of overfitting and improve generalisability (21). It has been applied to detect abnormal satellite engineering parameters, personal credit risk assessment, and urban water resources (22).

However, there are a limited number of studies on using ML for predicting health conditions of the older adults, and there are no studies on predicting their physical frailty status. We aimed to employ two supervised ML methods, RF and XGBoost, to explore three physical frailty assessment indices and construct prediction models. The physical frailty assessment indices were the 5-Item fatigue, resistance, ambulation, illness, and loss of weight (FRAIL) scale; Cardiovascular Health Study (CHS) index; and Study of Osteoporotic Fractures (SOF) index.

## Materials and methods

### Participants

The participants were included after obtaining informed consent and approval from the Institutional Review Board. We randomly selected community residents from three urban districts in Kaohsiung City, and randomly selected participants according to the proportion of the population over 60 years old. Participants were included to this study after informed consent. The inclusion criteria were: (1) aged 60 years and above, (2) ability to respond to a questionnaire, and (3) allowing for a physical assessment. The exclusion criteria were: (1) suffering from a mental disability or psychological disease, (2) unwillingness to provide informed consent and inability to cooperate with the study, and (3) acute hospitalization within the 3 months prior to the study. From April–October 2022, 445 participants were recruited for the study. This study was approved by the Kaohsiung Medical University Hospital Institutional Review Board [IRB number: KMUHIRB-E(I)-20220048].

### Measurements and questionnaire

All the participants were assessed through one-to-one interviews. After they completed the questionnaire and physical frailty assessment, we obtained their demographic characteristics, including sex, age, living environment, education level, and smoking and drinking habits. Elementary school education or no education was considered “low education.” The participants’ past histories were documented using their medical records obtained from their National Health Insurance cards. Physical examinations of height, weight, and blood pressure were also performed. The assessment indices for physical frailty included the (1) 5-Item FRAIL, (23) (2) CHS (Fried’s Frailty Phenotype), (24) and (3) SOF (25). Two researchers independently entered the data and confirmed their accuracy.

### Three tools for physical frailty assessment

The Geriatric Advisory Panel developed the 5-Item FRAIL scale, which comprises five items: (1) exhaustion, (2) weakness, (3) slowness while walking, (4) low activity, and (5) weight loss. Two items—fatigue and weight loss—were considered biological factors; another two—resistance and ambulation—were considered functional factors; and the last item was considered to involve deficit accumulation because of illness. The 5-Item FRAIL scale categorizes participants’ health statuses based on their scores as physical frail (3–5), physical pre-frail (1–2), and physical non-frail (0) (23).

The CHS index, a biological model of physical frailty, comprises five components: (1) unintentional weight loss, (2) feeling of exhaustion, (3) decreased physical activity, (4) slow walking speed, and (5) weakness, which are also used to classify health statuses based on scores as physical frail (3–5), physical pre-frail (1–2), and physical non-frail (0) (9, 24).

The SOF index comprises two factors with three components: (1) inability to complete five chair rises or suffering from weight loss, representing biological factors, and (2) reduced energy levels, representing a functional factor, which are also used to classify health statuses based on scores as physical frail (2–3), physical pre-frail (1), and physical non-frail (0) (25).

### Machine learning

The RF algorithm, developed by Breiman in 2001, (17) is an ensemble learning bagging algorithm (26). RF involves random sampling of the original training dataset, creating a new classifier for each sample, (27) and voting on the results generated by each classifier. The result is determined by voting on the results generated by each classifier, and the category with the largest number of votes constitutes the final result (28). RF requires minimal pruning and has no overfitting risk. Furthermore, it has high tolerance for outliers and noise, high adaptability to new samples, and good stability. Therefore, RF is suitable for parallel computing, even for high-dimensional data, with faster training speed and higher computing performance (29). The RF decision tree is built by selecting a feature at the root node and partitioning the training dataset into subsets of values of the selected feature (30). The information gain (IG) for partitioning training data y into subsets ($y_{i}$) is calculated as follows Equation (1):


(1) $$IG=-\underset{i}{\sum }\frac{\left|y_{i}\right|}{\left|y\right|}E\left(y_{i}\right)$$

where $E\left(y_{i}\right)$ is the entropy of set$y_{i}$and is calculated as Equation (2):


(2) $$E\left(y_{i}\right)=-\overset{n}{\underset{j=1}{\sum }}P_{j}\log_{2}\left(P_{j}\right)$$

The XGBoost algorithm, developed by Chen, (20) can be applied to handle regression and classification problems (31). It originated from the gradient boosting decision tree algorithm, which was modified to improve its generalisability and convergence rate (32). Boosting is an ensemble learning algorithm that converts weak classifier iterative learning into a strong classifier algorithm (32). It produces a new decision tree at each iteration based on the residuals of the previous one (33). XGBoost enhances the regularization of the loss function as a whole to create an objective function and improve the performance of the algorithm, (34) which is described in Equation (3).


(3) $$J\left(\theta \right)=L\left(\theta \right)+R\left(\theta \right)$$

where θis the parameter for data training, L is the loss function, and R is the regularization. Because the decision tree is the base model, the output of model $\hat{y_{i}}$ is an ensemble of k decision trees and is computed as follows Equation (4):


(4) $$\hat{y_{i}}=\overset{k}{\underset{i=1}{\sum }}f_{k}\left(\chi_{i}\right),f_{k}\in F$$

where $\chi_{i}$ is the i^th^ sample in the training set and F is the decision tree value.

Loss function L is calculated as follows Equation (5):


(5) $$L=\overset{n}{\underset{i}{\sum }}\left(\hat{y_{i}},y_{i}\right)+\underset{k}{\sum }\Omega \left(f_{k}\right)$$

(6) $$\Omega \left(f_{k}\right)=\Upsilon Τ+\frac{1}{2}\lambda {\left|w\right|}^{2}$$

where T is the number of trees in the leaf and w is the leaf weight in Equation (6).

### Evaluation metrics

To evaluate the performances of the RF and XGBoost algorithms for classifying the participant assessments on the 5-Item FRAIL, CHS, and SOF indices into robust, pre-frail, and frail, we employed the common evaluation indicators for ML classification: Accuracy (Equation 7), Precision (Equation 8), Recall (Equation 9), and F1 score (Equation 10): (35).


(7) $$\mathrm{Accuracy}=\frac{TP+TN}{TP+FP+TN+FN}\times 100$$

(8) $$\mathrm{Precision}=\frac{TP}{TP+FP}\times 100$$

(9) $$\mathrm{Recall}=\frac{TP}{TP+FN}\times 100$$

(10) $$F1-\mathrm{score}=\frac{\left(\mathrm{Precision}\times \mathrm{Recall}\right)\times 2}{\mathrm{Precision}+\mathrm{Recall}}$$

### Shapley additive explanations (SHAP)

SHAP, proposed by Lundberg and Lee in 2017, (36) is a framework for a unified interpretation of different ML prediction models (37). It is a Shapley value based on game theory (38) that explains the impact of each feature on an ML prediction (39). It is useful for both single- and full-feature interpretability; therefore, it can be used for the entire dataset to explain the influence of each feature on the prediction (39).

### Statistics

Descriptive statistics were used to analyse the mean and dispersion of continuous variables, including age and physical frailty scores. Numbers and proportions were used to evaluate categorical variables such as sex, smoking, and alcohol consumption. Furthermore, the participants were divided into groups according to their physical frailty status. The scores for the 5-Item FRAIL, CHS, and SOF indices were classified into physical non-frail, physical pre-frail, and physical frail groups. Statistical analyses were performed using IBM SPSS version 20 and Python (version 3.8.8).

## Results

### Demographic characteristics

In total, 445 participants satisfied the inclusion criteria. They were classified into physical non-frail, physical pre-frail, and physical frail groups according to their scores on the three indices and their demographic characteristics were determined, as listed in Table 1. According to the 5-Item FRAIL scale, 196 (44.04%), 184 (41.35%), and 65 (14.61%) participants were classified into physical non-frail, physical pre-frail, and physical frail groups, respectively. According to the CHS index, 144 (32.36%), 145 (32.58%), and 156 (35.06%) participants were classified into physical non-frail, physical pre-frail, and physical frail groups, respectively. According to the SOF index, 230 (51.69%), 152 (34.16%), and 63 (14.15%) participants were classified into physical non-frail, physical pre-frail, and physical frail groups, respectively. The average age of the participants was 68.75 years, and their average body mass index (BMI) was 25.39. They comprised 163 men (36.63%) and 282 women (63.37%). Moreover, 38.20% (170) had low levels of education (elementary school only or no education), 9.66% (40) lived alone, 4.49% (19) were smokers, and 10.11% (41) consumed alcohol.

**Table 1:** Demographic characteristics for model prediction according to the three physical frailty indices: 5-Item FRAIL, CHS, and SOF.

	5-Item FRAIL scale			CHS index			SOF index		
	Physical non-frail (*n* = 196)	Physical pre-frail (*n* = 184)	Physical frail (*n* = 65)	Physical non-frail (*n* = 144)	Physical pre-frail (*n* = 145)	Physical frail (*n* = 156)	Physical non-frail (*n* = 230)	Physical pre-frail (*n* = 152)	Physical frail (*n* = 63)
	*n*(%)/Mean ± SD	*n*(%)/Mean ± SD	*n*(%)/Mean ± SD	*n*(%)/Mean ± SD	*n*(%)/Mean ± SD	*n*(%)/Mean ± SD	*n*(%)/Mean ± SD	*n*(%)/Mean ± SD	*n*(%)/Mean ± SD
Sex (male)	74	60	29	54	47	62	88	52	23
Age (years)	65.80 ± 5.30	69.20 ± 6.59	76.28 ± 8.38	66.04 ± 4.35	66.00 ± 6.49	73.78 ± 7.38	65.87 ± 4.84	70.96 ± 7.82	73.88 ± 8.40
elementary school or no education	39	84	46	22	46	102	56	75	39
Live alone (yes)	17	19	7	13	14	16	19	17	7
No elevator in the house (yes)	167	158	56	121	125	136	196	131	55
No religion (yes)	25	18	7	20	20	10	28	16	6
Smoke (yes)	11	6	3	9	5	6	13	4	3
Alcohol (yes)	26	14	5	16	16	13	31	10	4
Lack of exercise (yes)	8	11	3	5	9	8	10	11	1
Hypertension (yes)	69	103	44	47	59	110	87	90	39
Diabetes mellitus (yes)	49	74	33	32	47	77	61	63	32
Hyperlipidemia (yes)	47	68	23	36	45	57	62	50	26
Cerebral vascular disease (yes)	5	12	5	5	3	14	8	10	4
Heart disease (yes)	24	27	22	18	17	39	30	30	14
Pulmonary disease (yes)	32	25	22	21	22	36	33	27	19
Liver disease (yes)	19	22	4	14	19	12	22	17	6
Urology disease (yes)	11	22	27	6	10	44	16	24	20
Malignancy (yes)	13	13	5	11	4	16	13	8	10
Sleep disorder (yes)	27	44	21	27	21	44	39	37	16
Neurological disease (yes)	1	5	1	1	2	5	1	5	2
Thyroid disease (yes)	25	25	3	19	20	14	26	20	7
Gastrointestinal disease (yes)	33	43	13	25	29	35	38	36	15
Hematological disease (yes)	2	1	2	2	0	3	3	0	2
Arthritis (yes)	22	36	14	16	25	31	33	22	17
Osteoporosis (yes)	17	28	8	15	17	21	23	17	13
Spine disorder (yes)	14	22	10	9	15	22	18	14	14
Rheumatic disease (yes)	3	4	0	4	1	2	5	2	0
Gout (yes)	3	4	11	3	3	12	6	5	7
Polypharmacy (yes)	5	23	24	3	9	41	16	22	15
Systolic blood pressure (mmHg)	142.29 ± 19.82	141.04 ± 19.63	150.37 ± 23.49	140.49 ± 18.81	143.28 ± 22.59	144.87 ± 19.76	141.09 ± 20.37	145.06 ± 20.81	144.53 ± 19.70
Diastolic blood pressure (mmHg)	82.39 ± 9.36	79.21 ± 10.10	79.00 ± 12.12	81.59 ± 9.32	82.30 ± 10.19	78.05 ± 10.54	81.67 ± 9.67	80.23 ± 10.34	77.44 ± 11.11
Body mass index (kg/m^2^)	25.13 ± 3.77	25.56 ± 3.80	25.64 ± 4.04	24.60 ± 3.46	25.72 ± 3.80	25.82 ± 4.07	25.08 ± 3.88	25.92 ± 3.77	25.22 ± 3.64

FRAIL, Fatigue, Resistance, Ambulation, Illness and Loss of Weight; CHS, Cardiovascular Health Study; SOF, Study of Osteoporotic Fracture; SD, standard deviation.

### ML algorithms: RF and XGBoost

**Figure 1:**
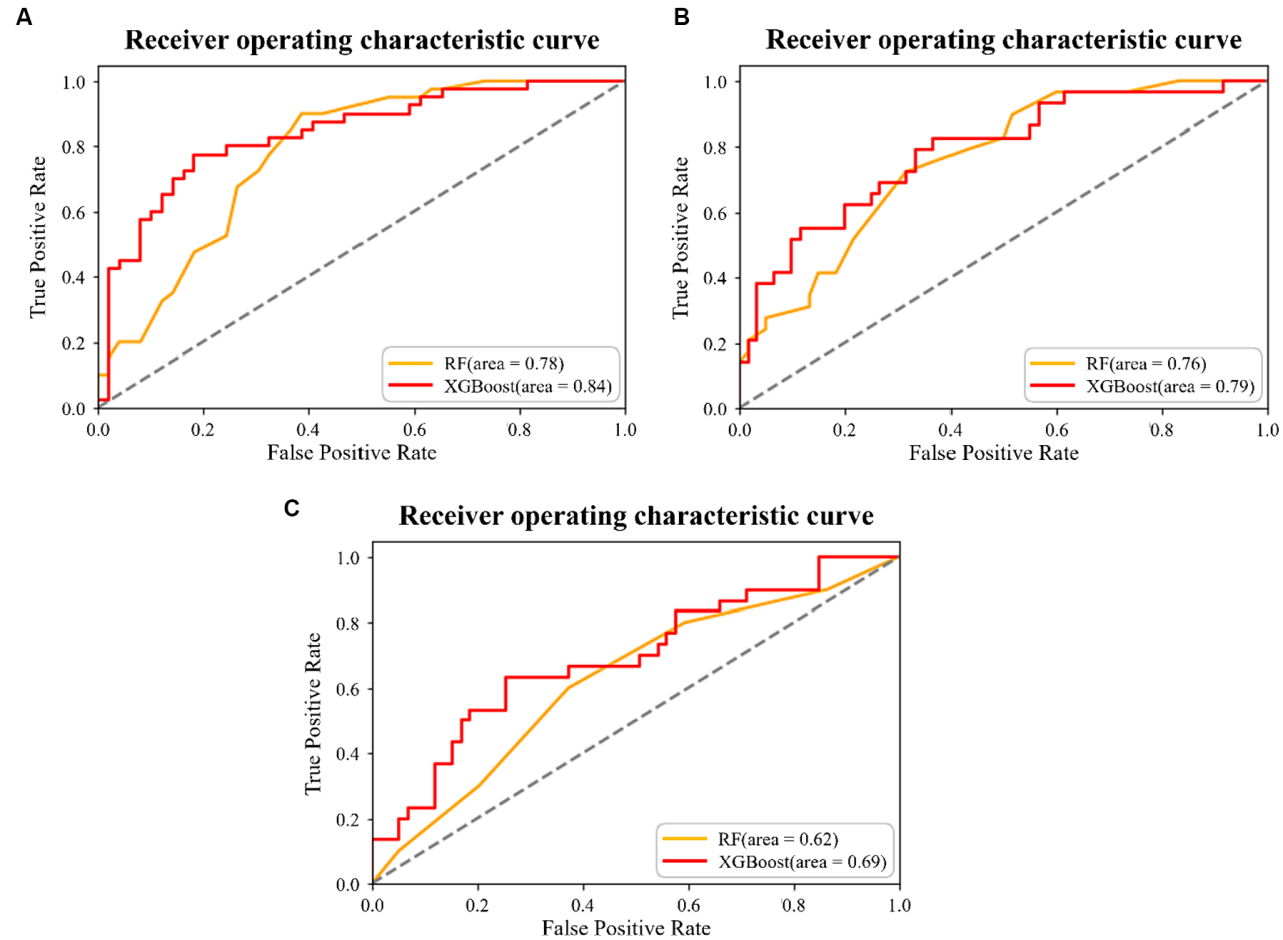
Results of the two machine learning algorithms for the **(A)** 5-Item Fatigue, Resistance, Ambulation, Illness, and Loss of Weight (FRAIL) scale prediction, **(B)** Cardiovascular Health Study (CHS) index prediction, and **(C)** Study of Osteoporotic Fracture (SOF) index prediction.

The XGBoost and RF predictions were compared based on accuracy, recall, precision, and F1 score. Compared with RF, XGBoost predicted the 5-Item FRAIL scale, CHS index, and SOF index with higher accuracy (Table 2). The receiver operating characteristic (ROC) curve was used to estimate model performance, with the ordinate and abscissa representing the frequencies of true and false positives, respectively. For the 5-Item FRAIL scale, the area under the ROC curve (AUC) of the RF algorithm was 0.78, and that of the XGBoost algorithm was 0.84, as shown in Figure 1A. For the CHS index, AUC of RF was 0.76, and that of the XGBoost was 0.79, as shown in Figure 1B. For the SOF index, AUC of RF was 0.62, and that of XGBoost was 0.69, as shown in Figure 1C. In summary, XGBoost had a better predictive ability than RF.

**Table 2:** Scores for the three physical frailty indices, 5-Item FRAIL, CHS, and SOF predicted using RF and XGBoost.

	RF	XGBoost
5-Item FRAIL scale		
Accuracy (%)	70.78	76.40
Recall (%)	75.31	73.48
Precision (%)	66.55	73.01
F1 score (%)	66.73	72.76
CHS index		
Accuracy (%)	68.53	70.78
Recall (%)	69.89	74.44
Precision (%)	68.04	70.38
F1 score (%)	64.23	68.22
SOF index		
Accuracy (%)	56.17	68.53
Recall (%)	52.59	62.72
Precision (%)	46.86	59.94
F1 score (%)	47.34	60.37

FRAIL, Fatigue, Resistance, Ambulation, Illness and Loss of Weight; CHS, Cardiovascular Health Study; SOF, Study of Osteoporotic Fracture; RF, random forest; XGBoost, extreme gradient boosting.

### Feature importance

**Figure 2:**
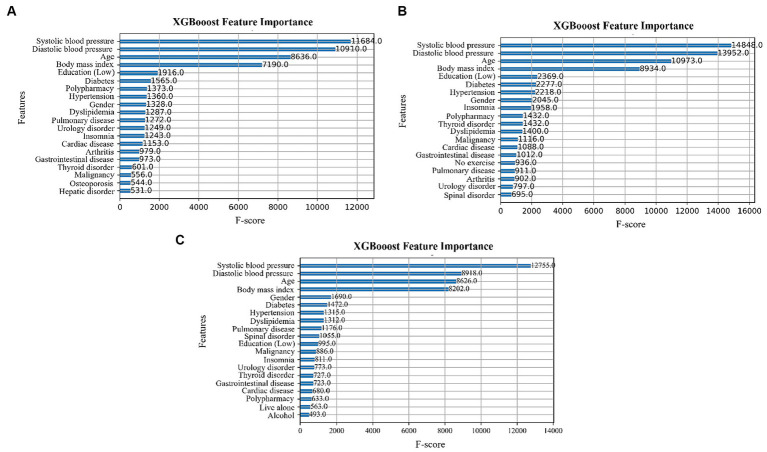
Feature importance for the **(A)** 5-Item FRAIL scale, **(B)** CHS index, and **(C)** SOF index.

Feature importance was determined using the XGBoost algorithm. The F-score indicates the number of times a feature is split during model training (42). The higher the score, the more important the feature and the greater its impact on the classification results (43). Figures 2A–C show the feature importance in the 5-Item FRAIL, CHS, and SOF indices, respectively. In all three, systolic blood pressure, diastolic blood pressure, age, and BMI have the top four F-score values.

### SHAP

**Figure 3:**
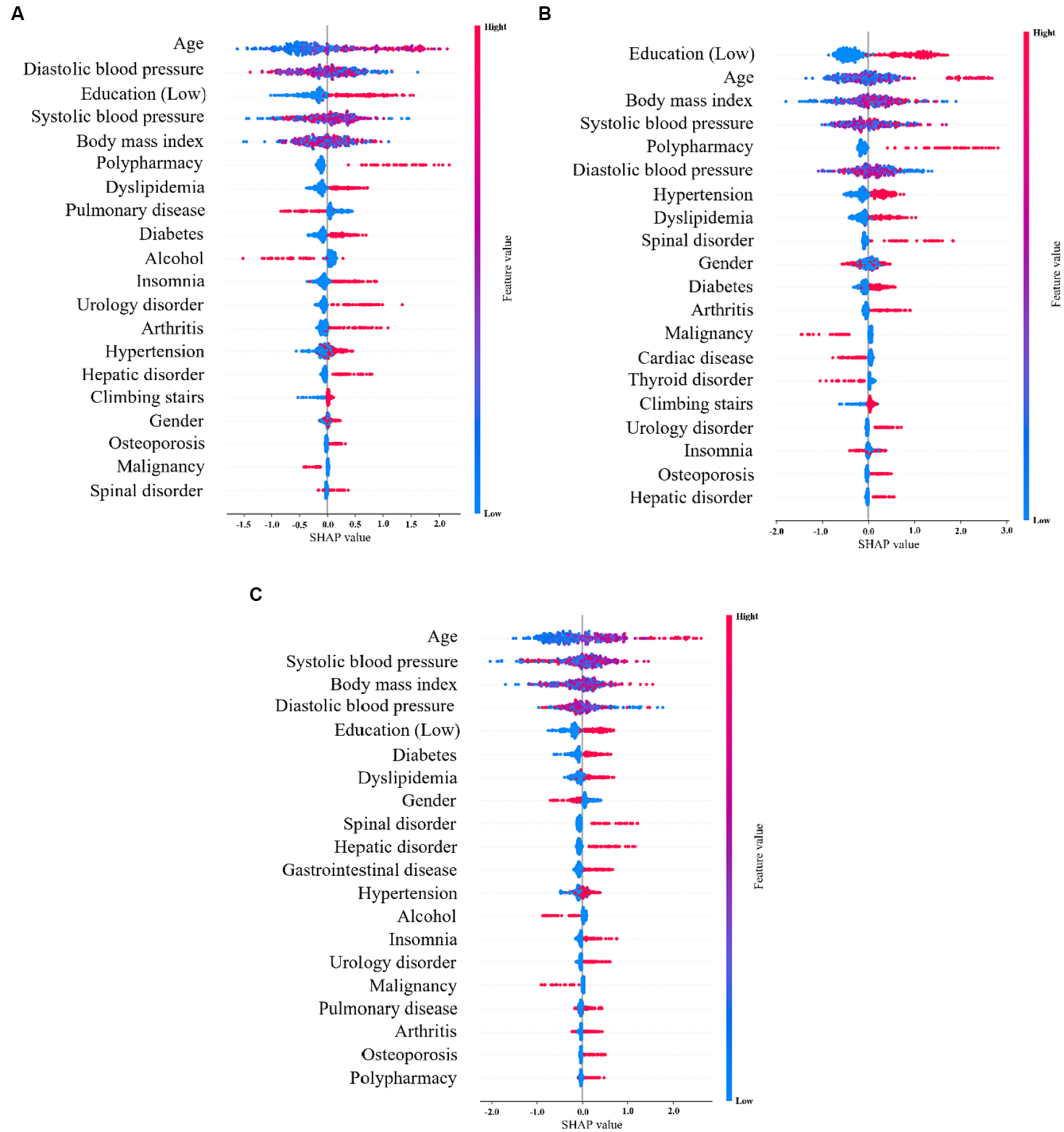
SHAP values for the **(A)** 5-Item FRAIL scale, **(B)** CHS index, and **(C)** SOF index.

SHAP shows the contribution of important features across the dataset. The x-axis represents the Shapley value and the y-axis represents the important features in the dataset, which are sorted according to their Shapley values. In the SHAP graph, the red points indicate that the value of the data is higher, and blue points indicate that the value of the data is lower. Figure 3A shows the SHAP values of the top 20 features in the 5-Item FRAIL scale, wherein the eigenvalues of age, diastolic blood pressure, systolic blood pressure, and BMI all affected the predicted value to some extent, and polypharmacy showed a positive correlation, indicating that the larger the feature value, the higher its contribution to the prediction. Figure 3B shows the SHAP values of the top 20 characteristics of the CHS index, where the eigenvalues of age, diastolic blood pressure, systolic blood pressure, and BMI affect the predicted value to some extent, and polypharmacy and urology disorders are positively correlated, indicating that the characteristics with larger values contribute more to the model prediction. Figure 3C shows the SHAP values of the top 20 SOF features. The eigenvalues of age, systolic blood pressure, BMI, and diastolic blood pressure affected the predicted value.

### Post-stratification of HTN

Table 3 shown the proportion of HTN or non-HTN in the three frailty assessments. Compared with physical non-frail population, HTN take significantly larger proportion in physical frail population in all three assessment classifications.

**Table 3:** The post-stratification of HTN and non-HTN in the three frailty assessments, 5-Item FRAIL scale, CHS index, and SOF index.

	5-Item FRAIL scale			*p*-value
	Physical non-frail	Physical pre-frail	Physical frail	
Non-HTN	127 (64.8%)	81 (44.0%)	21 (32.3%)	<0.001*
HTN	69 (35.2%)	103 (56.0%)	44 (67.7%)	
	CHS index			
	Physical non-frail	Physical pre-frail	Physical frail	
Non-HTN	97 (67.4%)	86 (59.3%)	46 (29.5%)	<0.001*
HTN	47 (32.6%)	59 (40.7%)	110 (70.5%)	
	SOF index			
	Physical non-frail	Physical pre-frail	Physical frail	
Non-HTN	143 (62.2%)	62 (40.8%)	24 (38.1%)	<0.001*
HTN	87 (37.8%)	90 (59.2%)	39 (61.9%)	

FRAIL, Fatigue, Resistance, Ambulation, Illness and Loss of Weight; CHS, Cardiovascular Health Study; SOF, Study of Osteoporotic Fracture.

^*^p-value < 0.05.

## Discussion

To compare RF and XGBoost, the same data were used for the training and testing evaluation. Overall, XGBoost performed better than RF. A significant difference was observed between high recall and low precision, as shown in Table 2. The recall rate is calculated by dividing the true positives by anything that should have been predicted as positive. Precision refers to the number of actual positives among the positive predictions, and a high recall rate indicates that the number of false positives are low, which is generally desirable. In summary, the XGBoost algorithm achieved a better prediction rate.

The exceptional predictive accuracy of XGBoost compared to Random Forest is the result of several unique techniques and features integral to XGBoost’s approach. Notably, its Gradient Boosting Framework allows for systematic improvements in predictions by specifically addressing errors from previous training rounds, employing gradient descent to reduce loss with each new addition (40). Additionally, XGBoost incorporates a regularization term in its objective function, which serves to prevent overfitting by penalizing overly complex models, thus fostering more generalizable and robust predictions. It also employs a sophisticated tree pruning method, which ensures the retention of only the most beneficial structures. Furthermore, XGBoost’s built-in routine for handling missing values, which intelligently decides the best course of action to minimize loss, significantly enhances its predictive capabilities (44). These combined features not only enhance XGBoost’s efficiency but also establish it as a formidable tool in machine learning competitions and applications where prediction accuracy is paramount.

The uniqueness of this study is that it employed ML to explore and address the characteristics of physical frailty predictions. The RF algorithm is a widely used ML algorithm in many fields (41) and has high accuracy, robustness, and the ability to handle high-dimensional data (30). It has been applied to the Minnesota Multiphasic Personality Inventory scale, and resulted in better classification and prediction (45). The XGBoost algorithm is a new ensemble learning method with an excellent implementation performance. Compared to other classifiers, XGBoost is anti-overfitting, highly efficient, entails low computational cost, and has better generalisability and accuracy compared to other ML algorithms (46, 47). The XGBoost algorithm has been previously applied to mental health prediction. Six ML algorithms were used to predict mental health using electronic medical records, of which XGBoost obtained the highest AUC value (48). Therefore, ML, especially the XGBoost algorithm, is better for classification and prediction of the three physical frailty indices: 5-Item FRAIL, CHS, and SOF.

Our study suggests that the 5-Item FRAIL is more aligned or similar to the SOF Index when it comes to classifying individuals who are physically frail. This implies that both tools might share common criteria or assess similar aspects of frailty, making them more interchangeable or comparable for identifying frail individuals. When it comes to classifying physical pre-frailty, the CHS Index is said to be closer to the SOF Index (49). This means that for identifying individuals who are not fully frail but have some signs of frailty (pre-frail), the CHS Index and SOF Index might share more similarities or provide more consistent classifications compared to other combinations of indices or scales. The result implies a comparison of the effectiveness or similarity of different frailty assessment tools, which is crucial for research, clinical practice, and policy-making, as identifying and managing frailty can help improve quality of life, reduce healthcare costs, and delay or prevent the progression to disability.

In this study, we used the SHAP tool and XGBoost algorithm to determine feature importance for a better understanding of these predictors. Figures 2, 3 show that among the top 20 important features, the influences of age, diastolic blood pressure, systolic blood pressure, and BMI on the prediction of the ML algorithms can be clearly understood. This indicates that a higher age is associated with higher physical frailty. For glioma grading, Cheng et al. applied the deep neural network model and SHAP tool, which not only shows the importance of every feature on the outcome but also indicates the influences of the associations between features on the predictions (50). For patients with severe COVID-19 intubation, Fleuren et al. applied the SHAP and found predictors of extubation failure, including ventilatory settings, inflammatory parameters, neurological status, and BMI (51). Hathaway et al. (52) conducted supervised learning through SHAP by identifying the most relevant and novel cardiac biomarkers for forecasting diabetes mellitus development, and discovered that this approach may be a potential guideline for investigating disease pathogenesis and discovering novel biomarkers in the future. For predicting infant autopsy outcome, Booth et al. used three models for model training, including decision tree, RF, and gradient boosting. Fundamental data items associated with determining the medical cause of death, including the most important items, such as age at death and cardiovascular and respiratory histological findings, were recognized using model feature importance, with the XGBoost algorithm being the most effective (53). The SHAP method and its feature importance classification can further assist clinicians in expanding their knowledge of the fundamental mechanisms by which predictors affect the output of ML models for health outcomes.

In our study, hypertension is recognized as one of the important predictive factors in the frailty among older adults. Studies have shown that hypertension can contribute to the development of frailty by affecting cardiovascular health, leading to impairments in physical function and an increased risk of adverse health outcomes (54). Research by Fried et al. (9) in the criteria for frailty, highlight the relationship between hypertension and frailty, suggesting that managing hypertension could be crucial in preventing or mitigating frailty in the older population. Our study represents the first instance of utilizing ML techniques to explore this domain, and remarkably, we have found results that align closely with those of previous studies.

This study had several limitations. First, it was a cross-sectional study that could only demonstrate associations and not infer causality. Further longitudinal studies are required to determine the causality between the possible risk factors and physical frailty. Second, we used self-reported questionnaires, and the results may have been influenced by recall biases such as memory, mood, or cognition. Third, ML models require a large amount of historical data for training to ensure that the model is not biased, (55) and it must be combined with datasets from other medical institutions to improve their predictive ability (56), such as Goh’s study, which aim to develop a predictive model for bacteremia in septic patients using machine learning methods, analysing data from an emergency department (57). Fourthly, the economic factor, a critical determinant that could significantly influence physical frailty through insufficient access to nutrition and healthcare, was omitted from the machine learning models. This oversight highlights the necessity of integrating economic considerations into future research. Incorporating this factor into subsequent studies will allow for a more comprehensive analysis, potentially uncovering deeper insights into the dynamics between economic status and physical frailty. Fifth, because the dataset is inherently predictive, when the sample size is small, models may face challenges. One of these challenges is the high sensitivity to outliers, which may overly emphasize anomalies in the samples, leading the ML model to believe that these outliers have a greater impact (52). Due to limitations in the dataset, the model may overfit to the training data, especially when using derived models like classification trees. This means that during training, the model may generate a branch for each patient sample, and such a complex model may not generalize well to new, (58) unseen data because it overly caters to the details and noise in the training data. Furthermore, training ML models is costly, and stakeholders, such as governments and major hospitals, must be persuaded, trained, and educated on ML applications; therefore, the adoption of ML algorithms is another challenge. These issues must be addressed to obtain the optimal gains in predictive accuracy (55). In light of these limitations encountered in this study, there are several promising avenues for deepening future research. Primarily, undertaking longitudinal studies emerges as a critical next step to establish causality between risk factors and physical frailty, moving beyond the associations observed in a cross-sectional framework. Additionally, future studies should consider employing objective measures alongside or in place of self-reported questionnaires to mitigate the impact of recall bias and enhance the reliability of data. The integration of economic factors into ML models is another vital area for exploration, aiming to capture the nuanced impacts of socioeconomic status on physical frailty. This inclusion promises a more rounded analysis and could reveal intricate dynamics that have been previously overlooked. Expanding the datasets for ML training by incorporating data from a variety of medical institutions will also be crucial in improving the models’ predictive accuracy and reducing bias. Lastly, addressing the challenges related to the cost and complexity of ML model training, as well as fostering stakeholder engagement, are essential steps for the broader adoption and application of ML in healthcare research. These focused directions not only aim to rectify the limitations of the current study but also pave the way for more comprehensive and impactful future research on physical frailty.

## Conclusion

This study demonstrated that two machine learning models are used for physical frailty assessing by the 5-item FRAIL scale, CHS index, and SOF index. XGBoost model is more precise predictive rate than RF model in all the three physical frailty models. Machine learning might be a useful instrument for early detection of physical frailty in the future. Furthermore, this study highlights the transformative potential of machine learning, especially the XGBoost algorithm’s efficacy in frailty assessments, for advancing early detection practices in healthcare. By integrating the XGBoost model, this research not only promises significant improvements in health care but also emphasizes the importance of such findings in informing health policy development. Furthermore, it offers practical guidance for healthcare professionals on leveraging these insights to enhance frailty management strategies for the aging population.

## Data availability statement

The raw data supporting the conclusions of this article will be made available by the authors, without undue reservation.

## Ethics statement

The studies involving humans were approved by Kaohsiung Medical University Hospital Institutional Review Board. The studies were conducted in accordance with the local legislation and institutional requirements. The participants provided their written informed consent to participate in this study.

## Author contributions

C-CY: Conceptualization, Data curation, Funding acquisition, Writing – original draft. P-HC: Formal analysis, Methodology, Software, Writing – original draft. C-HY: Conceptualization, Formal analysis, Methodology, Resources, Writing – review & editing. C-YD: Investigation, Writing – review & editing. K-HL: Formal analysis, Investigation, Writing – review & editing. T-HC: Investigation, Writing – review & editing. H-YC: Conceptualization, Formal analysis, Supervision, Writing – review & editing. C-HK: Supervision, Writing – review & editing.
